# K_1.65_V_1.78_W_0.22_O_2_(AsO_4_)_2_

**DOI:** 10.1107/S1600536809033133

**Published:** 2009-08-29

**Authors:** Sabrina Belkhiri, Mohammed Kars, Djillali Mezaoui

**Affiliations:** a Université Houari-Boumedienne, Faculté de Chimie, Laboratoire Sciences des Matériaux USTHB, BP 32 El-Alia, 16111 Bab-Ezzouar, Alger, Algérie

## Abstract

The title potassium vanadium tungsten bis­(arsenate oxide) was synthesized by a solid-state reaction at 973 K. The crystal structure is isotypic with KVOPO_4_ and contains a [*M*VAs_2_O_10_]_∞_ framework built up from single *M*O_6_ (*M* = V+W) octa­hedra sharing corners with single VO_6_ octa­hedra and AsO_4_ tetra­hedra. This structure shows the existence of infinite [VAsO_8_]_∞_ and [*M*AsO_8_]_∞_ chains running along the *a* and *c* directions, respectively. All atoms are located on general positions. The metal position *M* is statistically occupied by 78% V and 22% W.

## Littérature associée

La phase KVOPO_4_ a été décrite en premier par Benhamada *et al.* (1991 ▶). Quant à l’étude de la propriété optique du composé KTiOPO_4_ elle a été réalisée par Phillips *et al.* (1990 ▶). Les structures isotypes arséniates de formulation *M*TiOAsO_4_ (*M* = métal alcalin) ont été obtenues par réaction à l’état solide (El Haidouri *et al.*, 1990 ▶; Nakagawa *et al.*, 1999 ▶; El Brahimi & Durand, 1986 ▶). Pour la synthèse par la voie hydro­thermale des composés isotypes de formulation *M*ScFAsO_4_ (*M* = Rb et Cs), voir Harrison & Phillips (1999 ▶).

## Partie expérimentale

### 

#### Données crystallines

K_1.65_V_1.78_W_0.22_O_2_(AsO_4_)_2_
*M* *_r_* = 505,2
Orthorhombique, 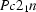

*a* = 6,5322 (7) Å
*b* = 10,7228 (9) Å
*c* = 13,0782 (5) Å
*V* = 916,04 (13) Å^3^
*Z* = 4
Mo *K*α radiation
μ = 12,70 mm^−1^
*T* = 293 K
0,07 × 0,04 × 0,03 mm

#### Collection de données

Diffractomètre Nonius KappaCCD
Correction d’absorption: Gaussian (*JANA2000*; Petříček *et al.*, 2000 ▶) *T*_min_ = 0,314, *T*_max_ = 0,564
13762 réflexions mesurées
3754 réflexions indépendantes
2938 réflexions avec *I* > 3σ(*I*)
*R*_int_ = 0,044

#### Affinement

*R*[*F* ^2^ > 2σ(*F* ^2^)] = 0,048
*wR*(*F* ^2^) = 0,064
*S* = 2,88
3754 réflexions
117 paramètres
5 contraintes
Δρ_max_ = 3,19 e Å^−3^
Δρ_min_ = −2,75 e Å^−3^
Structure absolute: Flack (1983 ▶), 1502 Friedel pairs
Paramètre Flack: 0,43 (3)

### 

Collection des données: *KappaCCD Software* (Nonius, 1998 ▶); affinement des paramètres de la maille: *DENZO* et *SCALEPACK* (Otwinowski & Minor, 1997 ▶); reduction des données: *DENZO* et *SCALEPACK*; méthode pour la solution de la structure: coordonnées prises de KVOPO_4_ (Benhamada *et al.*, 1991 ▶); programme(s) pour l’affinement de la structure: *JANA2000* (Petříček *et al.*, 2000 ▶); graphisme moléculaire: *ATOMS* (Dowty, 1994 ▶) et *GRETEP* (Laugier & Bochu, 2002 ▶); logiciel utilisé pour préparer le matériel pour publication: *JANA2000*.

## Supplementary Material

Crystal structure: contains datablocks global, I. DOI: 10.1107/S1600536809033133/br2112sup1.cif

Structure factors: contains datablocks I. DOI: 10.1107/S1600536809033133/br2112Isup2.hkl

Additional supplementary materials:  crystallographic information; 3D view; checkCIF report

## Figures and Tables

**Fig. 1.**
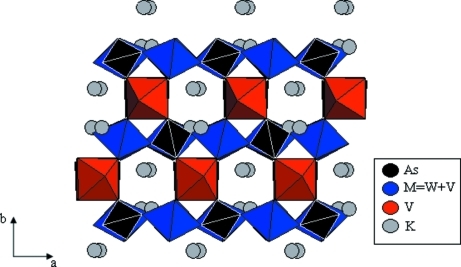
Projection de la structure K1.65V1.78W0.22O2(AsO4)2 selon c montrant la chaîne infinie [MAsO8]∞ (M = W+V) allongée suivant la direction a.

**Fig. 2.**
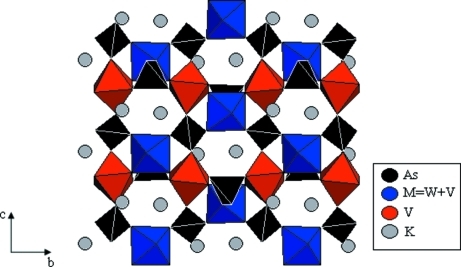
Projection de la structure K1.65V1.78W0.22O2(AsO4)2 selon a montrant la chaîne infinie [VAsO8]∞ (M = W+V) allongée suivant la direction c.

**Fig. 3.**
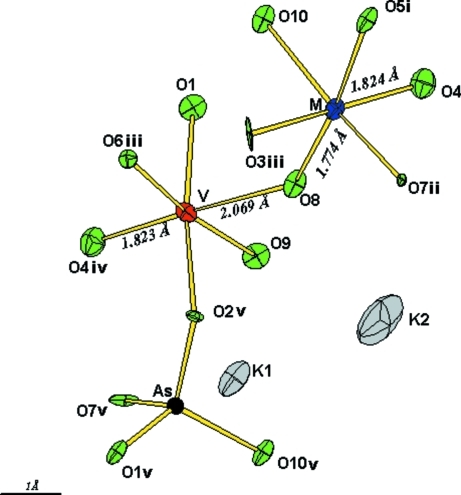
Fragment de la structure K1.65V1.78W0.22O2(AsO4)2. Les ellipsoïdes d'agitation thermique ont 60% de probabilité. codes de symétrie (i) x + 1/2 y + 1/2 - z + 3/2 (ii) x y z (iii) -x + 1/2 y z + 1/2 (iv) x - 1/2 y - 1/2 - z + 3/2 (v) x + 1/2 y - 1/2 - z + 3/2

**Fig. 4.**
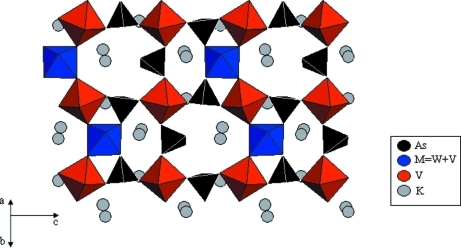
Projection de la structure K1.65V1.78W0.22O2(AsO4)2 selon la couche (110) montrant les chaînes purement octaédriques constituées de polyèdres alternés MO6 et VO6.

## supplementary crystallographic information

### Comment

Les composés de la famille KTiPO_5_ sont essentiellement connus pour leurs propriétés physiques en particulier la propriété optique non linèaire (Phillips *et al.*, 1990; Harrison & Phillips, 1999; El Haidouri *et al.*, 1990). Pour cette raison de nombreux composés isostructuraux à cette famille ont été synthétisés et étudiés (El Haidouri *et al.*, 1990; Nakagawa *et al.*, 1999; El Brahimi & Durand, 1986). Notre intérêt s'est porté sur les arséniates à base de vanadium dans le but de synthétiser des composés pouvant présenter des propriétés catalytiques, d'échange ioniques, d'optique non linèaire et de conduction ionique ainsi que des propriétés de transport. L'étude du système K–V–W–As–O nous a permis d'isoler une phase monocristalline de composition K_1.65_V_1.78_W_0.22_O_2_(AsO_4_)_2_ isotype à KVOPO_4_ (Benhamada *et al.*, 1991) par l'introduction du tungstène et de l'arsenic. La charpente [MVAs_2_O_10_]_∞_ (*M* = 78% V et 22% W) est constituée d'octaèdres MO_6_, VO_6_ et de tétraèdres AsO_4_ reliés par les sommets, formant des chaînes infinies [MAsO_8_]_∞_ (Fig. 1) et [VAsO_8_]_∞_ (Fig. 2) allongées respectivement suivant les directions *a* et *c*. Deux chaînes [VAsO_8_]_∞_ sont reliées entre elles par une chaîne [MAsO_8_]_∞_ et *vice versa*. Cette charpente [MVAs_2_O_10_]_∞_ délimite ainsi deux sortes de tunnels pseudo hexagonaux, dirigés selon *a* et *c*, dans lesquels sont localisés les ions potassium. Le premier type est construit par deux octaèdres VO_6_, deux octaèdres MO_6_ et deux tétraèdres AsO_4_ (Fig. 1), quant au second tunnel il est formé par deux octaèdres VO_6_, un seul octaèdre MO_6_ et de trois tétraèdres AsO_4_ (Fig. 2). Ces tunnels ont été déjà observés dans les composés de la famille KTiPO_5_. Les tétraèdres AsO_4_ ont la géométrie habituelle des monoarséniates avec des distances As—O (1,664 (7)–1,709 (7) Å), semblables à celles observées dans ATiOAsO_4_ (A = K, Rb, Cs et Tl) (1,63 (2)–1,76 (2) Å) (El Haidouri *et al.*, 1990; El Brahimi & Durand, 1986). Les octaèdres VO_6_ sont caractérisés par des distances V—O (1,823 (7)–2,069 (7) Å) semblables à celles observées dans le composé KVOPO_4_ (1,653 (4)–2,185 (4) Å) (Benhamada *et al.*, 1991) avec une courte et une longue distance de 1,823 (7) Å et 2,069 (8) Å respectivement ainsi que quatre distances intermédiaires (de 1,944 (7) à 2,006 (5) Å) correspondant aux atomes d'oxygènes communs avec les tétraèdres AsO_4_ (Fig. 3). Ce n'est pas le cas des octaèdres MO_6_ (*M* = W+V), où on observe deux courtes distances de 1,774 (7) Å et 1,824 (8) Å correspondant aux oxygènes communs avec les octaèdres VO_6_. Enfin, l'assemblage alterné de polyèdres VO_6_ et MO_6_ (*M* = V+W) (Fig. 4) conduit à la formation de chaînes octaédriques infinies [MO_3_]_∞_, qui laissent présager une certaine conduction électronique.

### Experimental

Les cristaux de K_1.65_V_1.78_W_0.22_O_2_(AsO_4_)_2_ ont été préparés à partir d'un mélange stoechiométrique de K_2_CO_3_, V_2_O_5_, WO_3_ et As_2_O_3_ chauffé dans un creuset en platine à 573 K pendant 24 heures. Une quantité adéquate du vanadium métallique est additionnée au mélange réractionnel ce dernier est ensuite placé dans un tube en quartz scellé sous vide et chauffé pendant 15 jours à 973 K. Une analyse qualitative au M.E.B confirme la présence des différents éléments chimiques attendus: K (32.93%), V (30.14%), As (31.86%) et W (5.07%), de composition proche de celle obtenue enfin d'affinement [K(at.%) = 29.20, V(at.%) = 31.50, As(at.%) = 35.40, W(at.%) = 3.89].

### Refinement

La structure a été déterminée par isotypie au composé KVOPO_4_ dans le groupe d'espace noncentrosymétrique P*c*2_1_n. En effet comme c'est le cas de la pluparts des composés de la famille KTiPO_5_, les essais d'affinements dans le groupe d'éspace centrosymétrique P*cmn* n'ont pas donnés de models de structure acceptables. Le site mixte *M* a été affiné avec une contrainte d'occupation totale égale à l'unité, donnant enfin d'affinement la formulation K_1.65_V_1.78_W_0.22_O_2_(AsO_4_)_2_. Le cristal étudié correspondant à  une macle d'inversion dont la fraction a été affiné à 0.43 (3).

### Crystal data

**Table:**

K_1.65_V_1.78_W_0.22_O_2_(AsO_4_)_2_	*F*(000) = 938
*M**_r_* = 505.2	*D*_x_ = 3.662 Mg m^−^^3^
Orthorhombic, *P**c*2_1_*n*	Mo *K*α radiation, λ = 0.71069 Å
Hall symbol: P -2n -2ac	Cell parameters from 25 reflections
*a* = 6.5322 (7) Å	θ = 5.8–36.0°
*b* = 10.7228 (9) Å	µ = 12.70 mm^−^^1^
*c* = 13.0782 (5) Å	*T* = 293 K
*V* = 916.04 (13) Å^3^	Block, black
*Z* = 4	0.07 × 0.04 × 0.03 mm

### Data collection

**Table:**

Nonius KappaCCD diffractometer	3754 independent reflections
Radiation source: fine-focus sealed tube	2938 reflections with *I* > 3σ(*I*)
graphite	*R*_int_ = 0.044
φ scans	θ_max_ = 36.0°, θ_min_ = 5.8°
Absorption correction: gaussian (Petříček *et al.*, 2000)	*h* = −10→10
*T*_min_ = 0.314, *T*_max_ = 0.564	*k* = −17→17
13762 measured reflections	*l* = −20→21

### Refinement

**Table:**

Refinement on *F*	(Δ/σ)_max_ = 0.0003
*R*[*F*^2^ > 2σ(*F*^2^)] = 0.048	Δρ_max_ = 3.19 e Å^−^^3^
*wR*(*F*^2^) = 0.064	Δρ_min_ = −2.75 e Å^−^^3^
*S* = 2.88	Extinction correction: B–C type 1 Gaussian isotropic (Becker & Coppens, 1974)
3754 reflections	Extinction coefficient: 2559
117 parameters	Absolute structure: Flack (1983), with how many Friedel pairs?
5 restraints	Flack parameter: 0.43 (3)
Weighting scheme based on measured s.u.'s *w* = 1/[σ^2^(*F*) + 0.0001*F*^2^]	

### Special details

**Table:**

Refinement. Refinement of *F*^2^ against ALL reflections. The weighted *R*-factor *wR* and goodness of fit *S* are based on *F*^2^, conventional *R*-factors are based on *F*, with *F* set to zero for negative *F*^2^. The threshold expression of *F*^2^ > n*σ(*F*^2^) is used only for calculating *R*-factors *etc*. and is not relevant to the choice of reflections for refinement. The program used for refinement, Jana2000, uses the weighting scheme based on the experimental expectations, see _refine_ls_weighting_details, that does not force *S* to be one. Therefore the values of *S* are usually larger then the ones from the *SHELX* program.

### Fractional atomic coordinates and isotropic or equivalent isotropic displacement parameters (Å^2^)

**Table:**

	*x*	*y*	*z*	*U*_iso_*/*U*_eq_	Occ. (<1)
V1	0.2630 (3)	0.2468 (2)	0.24971 (13)	0.0168 (3)	
W	0.50139 (17)	0.5	0.87349 (4)	0.00878 (14)	0.220 (2)
V2	0.50139 (17)	0.5	0.87349 (4)	0.00878 (14)	0.780 (2)
As	0.17664 (8)	0.25354 (16)	0.50018 (8)	0.00581 (11)	
As1	0.00101 (18)	0.50644 (9)	0.81691 (5)	0.00690 (13)	
O6	0.3321 (10)	0.2386 (6)	0.3990 (5)	0.0087 (13)	
O8	0.4578 (13)	0.3816 (6)	0.7822 (6)	0.0134 (7)	
K1	0.3033 (5)	0.5700 (3)	0.6047 (2)	0.0213 (8)	0.642 (9)
K2	0.7220 (5)	0.3138 (4)	0.6181 (3)	0.0639 (13)	
O4	0.5533 (11)	0.6264 (6)	0.7830 (6)	0.0116 (7)	
O7	−0.2009 (11)	0.4750 (6)	0.8937 (5)	0.0116 (7)	
O1	0.0429 (12)	0.3840 (6)	0.7363 (6)	0.0113 (12)	
O3	0.0142 (13)	0.3730 (6)	0.4904 (6)	0.0134 (7)	
O5	0.0306 (13)	0.1243 (6)	0.5126 (6)	0.0134 (7)	
O2	−0.0408 (12)	0.6253 (6)	0.7377 (7)	0.0160 (18)	
O9	0.3212 (11)	0.2750 (7)	0.6060 (5)	0.0113 (12)	
O10	0.2004 (10)	0.5412 (6)	0.8927 (5)	0.0116 (7)	

### Atomic displacement parameters (Å^2^)

**Table:**

	*U*^11^	*U*^22^	*U*^33^	*U*^12^	*U*^13^	*U*^23^
V1	0.0307 (7)	0.0156 (5)	0.0042 (4)	−0.0159 (5)	−0.0026 (4)	0.0014 (4)
W	0.0070 (2)	0.0118 (2)	0.0075 (3)	−0.00182 (17)	−0.0009 (4)	−0.0018 (4)
V2	0.0070 (2)	0.0118 (2)	0.0075 (3)	−0.00182 (17)	−0.0009 (4)	−0.0018 (4)
As	0.0070 (2)	0.00636 (17)	0.0040 (2)	−0.0010 (4)	0.0017 (4)	0.00093 (18)
As1	0.0054 (2)	0.00608 (19)	0.0092 (3)	0.00066 (15)	−0.0001 (5)	−0.0006 (3)
O6	0.008 (2)	0.011 (2)	0.007 (3)	0.0035 (19)	−0.0005 (18)	0.0001 (19)
O8	0.0123 (14)	0.0181 (11)	0.0099 (13)	−0.0028 (14)	−0.0004 (17)	0.0051 (9)
K1	0.0174 (13)	0.0400 (16)	0.0066 (13)	−0.0027 (11)	−0.0056 (10)	0.0009 (11)
K2	0.0193 (11)	0.086 (3)	0.087 (3)	−0.0147 (14)	0.0165 (16)	−0.0306 (19)
O4	0.0061 (10)	0.0160 (11)	0.0128 (13)	−0.0011 (9)	0.0026 (16)	0.0031 (14)
O7	0.0061 (10)	0.0160 (11)	0.0128 (13)	−0.0011 (9)	0.0026 (16)	0.0031 (14)
O1	0.012 (2)	0.018 (2)	0.004 (2)	0.0023 (15)	−0.0047 (17)	−0.0012 (16)
O3	0.0123 (14)	0.0181 (11)	0.0099 (13)	−0.0028 (14)	−0.0004 (17)	0.0051 (9)
O5	0.0123 (14)	0.0181 (11)	0.0099 (13)	−0.0028 (14)	−0.0004 (17)	0.0051 (9)
O2	0.018 (3)	0.012 (2)	0.018 (4)	0.012 (2)	0.005 (3)	0.007 (2)
O9	0.012 (2)	0.018 (2)	0.004 (2)	0.0023 (15)	−0.0047 (17)	−0.0012 (16)
O10	0.0061 (10)	0.0160 (11)	0.0128 (13)	−0.0011 (9)	0.0026 (16)	0.0031 (14)

### Geometric parameters (Å, °)

**Table:**

V1—W^i^	3.461 (2)	As—K2^vii^	3.407 (4)
V1—V2^i^	3.461 (2)	As—O4^viii^	3.251 (9)
V1—As	3.3257 (17)	As—O7^ix^	3.410 (7)
V1—As^ii^	3.2867 (17)	As—O1	3.497 (8)
V1—As1^iii^	3.222 (2)	As—O3	1.671 (7)
V1—As1^ii^	3.301 (2)	As—O5	1.688 (7)
V1—O6	2.006 (5)	As—O2^iii^	3.520 (8)
V1—O8^ii^	2.069 (7)	As—O9	1.695 (7)
V1—O4^i^	1.823 (7)	As—O10^ii^	3.474 (7)
V1—O7^iii^	3.494 (7)	As1—O6^v^	3.243 (6)
V1—O1^ii^	1.944 (7)	As1—O8	3.294 (7)
V1—O2^iii^	1.960 (7)	As1—K1	3.475 (3)
V1—O9^ii^	1.979 (7)	As1—O4^vii^	3.224 (7)
W—As^iv^	3.3815 (19)	As1—O7	1.685 (7)
W—As^v^	3.3284 (19)	As1—O1	1.709 (7)
W—As1	3.3521 (18)	As1—O2	1.664 (7)
W—As1^vi^	3.3470 (18)	As1—O9^x^	3.284 (7)
W—O6^v^	3.557 (6)	As1—O10	1.684 (6)
W—O8	1.774 (7)	O6—K1^i^	2.998 (7)
W—O4	1.824 (8)	O8—K1	3.240 (8)
W—O7^vi^	1.980 (7)	O8—K2	2.854 (8)
W—O3^v^	2.047 (7)	K1—O4	2.911 (9)
W—O5^iv^	2.014 (7)	K1—O1	3.132 (8)
W—O10	2.034 (7)	K1—O3	3.181 (8)
V2—As^iv^	3.3815 (18)	K1—O5^xi^	2.718 (8)
V2—As^v^	3.3284 (18)	K1—O2	2.903 (8)
V2—As1	3.3521 (13)	K1—O9	3.147 (8)
V2—As1^vi^	3.3470 (13)	K1—O10^ii^	2.775 (6)
V2—O6^v^	3.557 (6)	K2—O4^ix^	3.228 (8)
V2—O8	1.774 (7)	K2—O7^ii^	3.414 (8)
V2—O4	1.824 (8)	K2—O1^vi^	2.715 (8)
V2—O7^vi^	1.980 (7)	K2—O3^vi^	2.619 (8)
V2—O3^v^	2.047 (7)	K2—O5^vi^	3.145 (8)
V2—O5^iv^	2.014 (7)	K2—O2^ix^	3.250 (8)
V2—O10	2.034 (7)	K2—O9	2.656 (7)
As—O6	1.676 (6)	K2—O10^ix^	2.937 (8)
As—O8^ii^	3.279 (8)		
			
O6—V1—O8^ii^	89.1 (3)	O9—As—O10^ii^	94.0 (3)
O6—V1—O4^i^	92.9 (3)	O6^v^—As1—K1	104.26 (12)
O6—V1—O7^iii^	120.7 (2)	O6^v^—As1—O4^vii^	134.43 (16)
O6—V1—O1^ii^	88.3 (3)	O6^v^—As1—O7	83.4 (2)
O6—V1—O2^iii^	93.5 (3)	O6^v^—As1—O1	58.1 (3)
O6—V1—O9^ii^	172.3 (3)	O6^v^—As1—O2	159.4 (3)
O8^ii^—V1—O4^i^	177.4 (3)	O6^v^—As1—O9^x^	142.85 (15)
O8^ii^—V1—O7^iii^	127.7 (2)	O6^v^—As1—O10	74.7 (2)
O8^ii^—V1—O1^ii^	86.7 (3)	O8—As1—K1	57.12 (14)
O8^ii^—V1—O2^iii^	86.1 (3)	O8—As1—O4^vii^	164.2 (2)
O8^ii^—V1—O9^ii^	83.8 (3)	O8—As1—O7	134.6 (3)
O4^i^—V1—O7^iii^	49.7 (3)	O8—As1—O1	56.8 (3)
O4^i^—V1—O1^ii^	95.1 (3)	O8—As1—O2	111.9 (3)
O4^i^—V1—O2^iii^	92.0 (3)	O8—As1—O9^x^	136.53 (17)
O4^i^—V1—O9^ii^	94.3 (4)	O8—As1—O10	58.4 (2)
O7^iii^—V1—O1^ii^	131.3 (3)	O4^vii^—As1—O7	56.8 (3)
O7^iii^—V1—O2^iii^	53.2 (2)	O4^vii^—As1—O1	111.7 (3)
O7^iii^—V1—O9^ii^	66.4 (2)	O4^vii^—As1—O2	57.6 (3)
O1^ii^—V1—O2^iii^	172.6 (3)	O4^vii^—As1—O9^x^	50.74 (17)
O1^ii^—V1—O9^ii^	88.4 (3)	O4^vii^—As1—O10	134.1 (3)
O2^iii^—V1—O9^ii^	88.9 (3)	O7—As1—O1	109.3 (3)
O6^v^—W—O8	52.8 (2)	O7—As1—O2	113.4 (3)
O6^v^—W—O4	138.9 (3)	O7—As1—O9^x^	74.0 (3)
O6^v^—W—O7^vi^	118.5 (2)	O7—As1—O10	107.5 (3)
O6^v^—W—O3^v^	51.6 (2)	O1—As1—O2	103.5 (4)
O6^v^—W—O5^iv^	120.6 (2)	O1—As1—O9^x^	157.9 (3)
O6^v^—W—O10	64.1 (2)	O1—As1—O10	114.2 (3)
O8—W—O4	97.2 (3)	O2—As1—O9^x^	57.0 (3)
O8—W—O7^vi^	98.4 (3)	O2—As1—O10	109.0 (3)
O8—W—O3^v^	91.3 (3)	O9^x^—As1—O10	84.3 (2)
O8—W—O5^iv^	173.4 (3)	O6^xii^—K1—O8	98.39 (18)
O8—W—O10	94.6 (3)	O6^xii^—K1—O4	56.08 (19)
O4—W—O7^vi^	90.3 (3)	O6^xii^—K1—O1	145.5 (2)
O4—W—O3^v^	169.5 (3)	O6^xii^—K1—O3	149.53 (19)
O4—W—O5^iv^	88.0 (3)	O6^xii^—K1—O5^xi^	119.1 (2)
O4—W—O10	96.0 (3)	O6^xii^—K1—O2	119.8 (2)
O7^vi^—W—O3^v^	82.3 (3)	O6^xii^—K1—O9	125.3 (2)
O7^vi^—W—O5^iv^	85.6 (3)	O6^xii^—K1—O10^ii^	93.41 (19)
O7^vi^—W—O10	164.8 (2)	O8—K1—O4	51.74 (18)
O3^v^—W—O5^iv^	84.0 (3)	O8—K1—O1	51.25 (18)
O3^v^—W—O10	89.4 (2)	O8—K1—O3	95.96 (19)
O5^iv^—W—O10	80.8 (3)	O8—K1—O5^xi^	142.1 (2)
O6^v^—V2—O8	52.8 (2)	O8—K1—O2	86.3 (2)
O6^v^—V2—O4	138.9 (3)	O8—K1—O9	50.10 (18)
O6^v^—V2—O7^vi^	118.5 (2)	O8—K1—O10^ii^	130.1 (2)
O6^v^—V2—O3^v^	51.6 (2)	O4—K1—O1	89.6 (2)
O6^v^—V2—O5^iv^	120.6 (2)	O4—K1—O3	147.2 (2)
O6^v^—V2—O10	64.1 (2)	O4—K1—O5^xi^	148.6 (2)
O8—V2—O4	97.2 (3)	O4—K1—O2	84.7 (2)
O8—V2—O7^vi^	98.4 (3)	O4—K1—O9	100.3 (2)
O8—V2—O3^v^	91.3 (3)	O4—K1—O10^ii^	145.5 (2)
O8—V2—O5^iv^	173.4 (3)	O1—K1—O3	61.2 (2)
O8—V2—O10	94.6 (3)	O1—K1—O5^xi^	91.4 (2)
O4—V2—O7^vi^	90.3 (3)	O1—K1—O2	51.88 (18)
O4—V2—O3^v^	169.5 (3)	O1—K1—O9	51.67 (18)
O4—V2—O5^iv^	88.0 (3)	O1—K1—O10^ii^	118.1 (2)
O4—V2—O10	96.0 (3)	O3—K1—O5^xi^	54.16 (19)
O7^vi^—V2—O3^v^	82.3 (3)	O3—K1—O2	87.7 (2)
O7^vi^—V2—O5^iv^	85.6 (3)	O3—K1—O9	50.68 (18)
O7^vi^—V2—O10	164.8 (2)	O3—K1—O10^ii^	57.2 (2)
O3^v^—V2—O5^iv^	84.0 (3)	O5^xi^—K1—O2	71.5 (2)
O3^v^—V2—O10	89.4 (2)	O5^xi^—K1—O9	104.8 (2)
O5^iv^—V2—O10	80.8 (2)	O5^xi^—K1—O10^ii^	57.1 (2)
O6—As—O8^ii^	60.7 (2)	O2—K1—O9	103.3 (2)
O6—As—O4^viii^	143.3 (3)	O2—K1—O10^ii^	127.9 (2)
O6—As—O7^ix^	96.0 (3)	O9—K1—O10^ii^	83.97 (19)
O6—As—O1	152.0 (2)	O8—K2—O4^ix^	105.3 (2)
O6—As—O3	113.1 (3)	O8—K2—O7^ii^	120.0 (2)
O6—As—O5	111.0 (3)	O8—K2—O1^vi^	88.5 (2)
O6—As—O2^iii^	54.5 (2)	O8—K2—O3^vi^	149.5 (3)
O6—As—O9	108.8 (3)	O8—K2—O5^vi^	151.0 (2)
O6—As—O10^ii^	67.8 (2)	O8—K2—O2^ix^	53.10 (19)
O8^ii^—As—O4^viii^	150.29 (17)	O8—K2—O9	58.7 (2)
O8^ii^—As—O7^ix^	141.90 (17)	O8—K2—O10^ix^	105.1 (2)
O8^ii^—As—O1	122.41 (17)	O4^ix^—K2—O7^ii^	133.4 (2)
O8^ii^—As—O3	56.6 (3)	O4^ix^—K2—O1^vi^	55.0 (2)
O8^ii^—As—O5	105.8 (3)	O4^ix^—K2—O3^vi^	85.3 (2)
O8^ii^—As—O2^iii^	47.58 (16)	O4^ix^—K2—O5^vi^	49.5 (2)
O8^ii^—As—O9	143.0 (3)	O4^ix^—K2—O2^ix^	74.43 (19)
O4^viii^—As—O3	103.5 (3)	O4^ix^—K2—O9	126.1 (2)
O4^viii^—As—O5	55.0 (3)	O4^ix^—K2—O10^ix^	55.25 (19)
O4^viii^—As—O2^iii^	122.96 (18)	O7^ii^—K2—O1^vi^	112.7 (2)
O4^viii^—As—O9	59.1 (3)	O7^ii^—K2—O3^vi^	50.0 (2)
O4^viii^—As—O10^ii^	141.36 (16)	O7^ii^—K2—O5^vi^	88.3 (2)
O7^ix^—As—O1	92.65 (16)	O7^ii^—K2—O2^ix^	142.3 (2)
O7^ix^—As—O3	149.2 (3)	O7^ii^—K2—O9	89.2 (2)
O7^ix^—As—O5	51.8 (3)	O7^ii^—K2—O10^ix^	116.94 (19)
O7^ix^—As—O2^iii^	94.61 (16)	O1^vi^—K2—O3^vi^	74.1 (2)
O7^ix^—As—O9	70.2 (3)	O1^vi^—K2—O5^vi^	86.3 (2)
O7^ix^—As—O10^ii^	153.03 (15)	O1^vi^—K2—O2^ix^	104.5 (2)
O1—As—O3	66.3 (3)	O1^vi^—K2—O9	147.0 (3)
O1—As—O5	95.2 (3)	O1^vi^—K2—O10^ix^	110.1 (2)
O1—As—O2^iii^	150.96 (15)	O3^vi^—K2—O5^vi^	54.4 (2)
O1—As—O10^ii^	93.27 (15)	O3^vi^—K2—O2^ix^	155.1 (2)
O3—As—O5	105.7 (3)	O3^vi^—K2—O9	136.3 (3)
O3—As—O2^iii^	94.6 (3)	O3^vi^—K2—O10^ix^	104.4 (2)
O3—As—O9	107.2 (4)	O5^vi^—K2—O2^ix^	100.8 (2)
O3—As—O10^ii^	55.3 (3)	O5^vi^—K2—O9	120.2 (2)
O5—As—O2^iii^	68.1 (3)	O5^vi^—K2—O10^ix^	50.97 (18)
O5—As—O9	110.9 (4)	O2^ix^—K2—O9	54.5 (2)
O5—As—O10^ii^	153.1 (3)	O2^ix^—K2—O10^ix^	51.97 (18)
O2^iii^—As—O9	157.2 (3)	O9—K2—O10^ix^	78.3 (2)
O2^iii^—As—O10^ii^	92.87 (15)		

Symmetry codes: (i) −*x*+1, *y*−1/2, −*z*+1; (ii) −*x*+1/2, *y*, *z*−1/2; (iii) −*x*, *y*−1/2, −*z*+1; (iv) *x*+1/2, *y*+1/2, −*z*+3/2; (v) −*x*+1/2, *y*, *z*+1/2; (vi) *x*+1, *y*, *z*; (vii) *x*−1, *y*, *z*; (viii) *x*−1/2, *y*−1/2, −*z*+3/2; (ix) *x*+1/2, *y*−1/2, −*z*+3/2; (x) *x*−1/2, *y*+1/2, −*z*+3/2; (xi) −*x*, *y*+1/2, −*z*+1; (xii) −*x*+1, *y*+1/2, −*z*+1.
